# Traditional Chinese medicine inspired dual-drugs loaded inhalable nano-therapeutics alleviated idiopathic pulmonary fibrosis by targeting early inflammation and late fibrosis

**DOI:** 10.1186/s12951-023-02251-0

**Published:** 2024-01-03

**Authors:** Meiling Zheng, Kai Liu, Lei Li, Cuiling Feng, Guanghao Wu

**Affiliations:** 1 Dongzhimen Hospital, Beijing University of Chinese Medicine, Beijing, 100010 China; 2 Peking University People’s Hospital, Beijing, 100032 China; 3 School of Materials Science & Engineering, Beijing Institute of Technology, Beijing, 100081 China; 4 Division of Pulmonary and Critical Care Medicine, Kunming Children’s Hospital, Kunming, 650000 China; 5 Department of Obstetrics and Gynecology, Peking Union Medical College Hospital, National Clinical Research Center for Obstetric & Gynecologic Diseases, Chinese Academy of Medical Sciences & Peking Union Medical College, Beijing, 100032 China

**Keywords:** Idiopathic pulmonary fibrosis, Astragaloside IV, Ligustrazine, Nanoparticles, Inhalation

## Abstract

Idiopathic pulmonary fibrosis (IPF) is a highly debilitating and fatal chronic lung disease that is difficult to cure clinically. IPF is characterized by a gradual decline in lung function, which leads to respiratory failure and severely affects patient quality of life and survival. Oxidative stress and chronic inflammation are believed to be important pathological mechanisms underlying the onset and progression of IPF, and the vicious cycle of NOX4-derived ROS, NLRP3 inflammasome activation, and p38 MAPK in pulmonary fibrogenesis explains the ineffectiveness of single-target or single-drug interventions. In this study, we combined astragaloside IV (AS-IV) and ligustrazine (LIG) based on the fundamental theory of traditional Chinese medicine (TCM) of “tonifying qi and activating blood” and loaded these drugs onto nanoparticles (AS_LIG@PPGC NPs) that were inhalable and could penetrate the mucosal barrier. Our results suggested that inhalation of AS_LIG@PPGC NPs significantly improved bleomycin-induced lung injury and fibrosis by regulating the NOX4-ROS-p38 MAPK and NOX4-NLRP3 pathways to treat and prevent IPF. This study not only demonstrated the superiority, feasibility, and safety of inhalation therapy for IPF intervention but also confirmed that breaking the vicious cycle of ROS and the NLRP3 inflammasome is a promising strategy for the successful treatment of IPF. Moreover, this successful nanoplatform is a good example of the integration of TCM and modern medicine.

**Supplementary Information:**

The online version contains supplementary material available at 10.1186/s12951-023-02251-0.

## Introduction

Pulmonary fibrosis (PF) is a pathological process that involves the damage and repair of lung tissue and is characterized by chronic inflammation, oxidative stress, immune activation, and fatty acid metabolism [1–4]. PF is both a common feature of idiopathic pulmonary fibrosis (IPF) and the pathological endpoint of various diseases, including long-term exposure to harmful particles, inflammation, infections, and autoimmune diseases [5]. The incidence of IPF has significantly increased in recent decades, ranging from 8 to 60 cases per 100,000 individuals [6–8]. In addition, the poor prognosis of IPF and secondary carcinoma occurrence in PF patients are disheartening. Oral administration of pirfenidone (PFD) or nintedanib is widely used to manage IPF [9]. however, these drugs exhibit obvious side effects, such as nausea, diarrhea, and gastrointestinal discomfort. In addition, the high costs and unsatisfactory therapeutic efficacy limit their widespread clinical application [10, 11]. Therefore, it is urgent to develop a suitable, potent, and low-toxicity treatment to intervene in the progression of IPF.

Small molecules from natural products are key sources of innovative drugs. Traditional Chinese medicine (TCM) has demonstrated enormous potential in drug development, and artemisinin, which is commended by a Nobel Prize, is an outstanding achievement in TCM development. Qi invigoration and blood activation are basic treatments for IPF. *A. membranaceus* and *L. chuanxiong* are not only a classic combination for Qi invigoration and blood activation but are also the main components of Buyanghuanwu Decoction (BYD). In several previous studies, BYD has shown excellent therapeutic effects on PF [12, 13]. Astragaloside IV (AS-IV) and ligustrazine (LIG) are the effective bioactive components of *A. membranaceus* and *L. chuanxiong*, respectively, and are considered to have good antifibrotic potential [14–19]; therefore, AS-IV and LIG are expected to be candidates for IPF treatment. However, the use of AS-IV and LIG is severely impaired by low water solubility, poor bioavailability, and limited lung targeting. Efficient and stable codelivery of AS-IV and LIG to the lungs would improve the management of PF, and this is the main purpose of the current study.

Inhalation therapy offers natural advantages for treating lung diseases. Inhaled drugs can be delivered directly to the lungs and reduce the adverse effects caused by oral administration or intravenous injection [20]. Moreover, inhalation therapy improves drug utilization, reduces hepatic first-pass metabolism, and avoids gastrointestinal malabsorption. In addition, inhalation requires lower effective doses than oral or intravenous routes and can be delivered noninvasively through aerosol inhalation [21]. However, inhalation therapy faces challenges with drug particle size requirements and lung clearance mechanisms. The most optimized aerodynamic diameters provide the greatest potential for deposition in the lungs, which are less than 5 μm and 3 μm in adults [22] and children [23], respectively. The clearance mechanisms in the lung, including mucus, cilia and local macrophages, are major factors that affect the rate of aerosol deposition in the lungs [24, 25]. Therefore, designing a drug with a suitable aerodynamic diameter is necessary for achieving the optimal deposition rate in the lungs and improving the efficacy of inhalation therapy.

To optimize the inhalation system and overcome the potential challenges, polylactic-co-glycolic acid (PLGA) particles can be modified with PEG as a carrier of natural compounds. The final PEG-PLGA has an external size, internal structure, and surface structure that are all at the nanoscale level (i.e., less than 100 nm), or the particle size must be less than 1000 nm. The particle size of PEG-PLGA determines its ability to penetrate the physiological barrier of the lungs. An in vivo study showed that rat alveolar macrophages (AMs) engulfed fewer particles < 1 μm in size than larger particles (1–5 μm) [26]. PEG-PLGA is relatively safe and promotes the biocompatibility, stability, and circulation time of several drugs in vivo. Moreover, NPs with surfaces modified by PEG avoid being taken up by AMs [27]. However, there has been no research on the combined application of these two compounds for inhalation therapy to treat IPF. Therefore, the use of PEG-PLGA as a drug delivery system to encapsulate AS-IV and LIG for IPF treatment is of great research value.

**Fig. 1:**
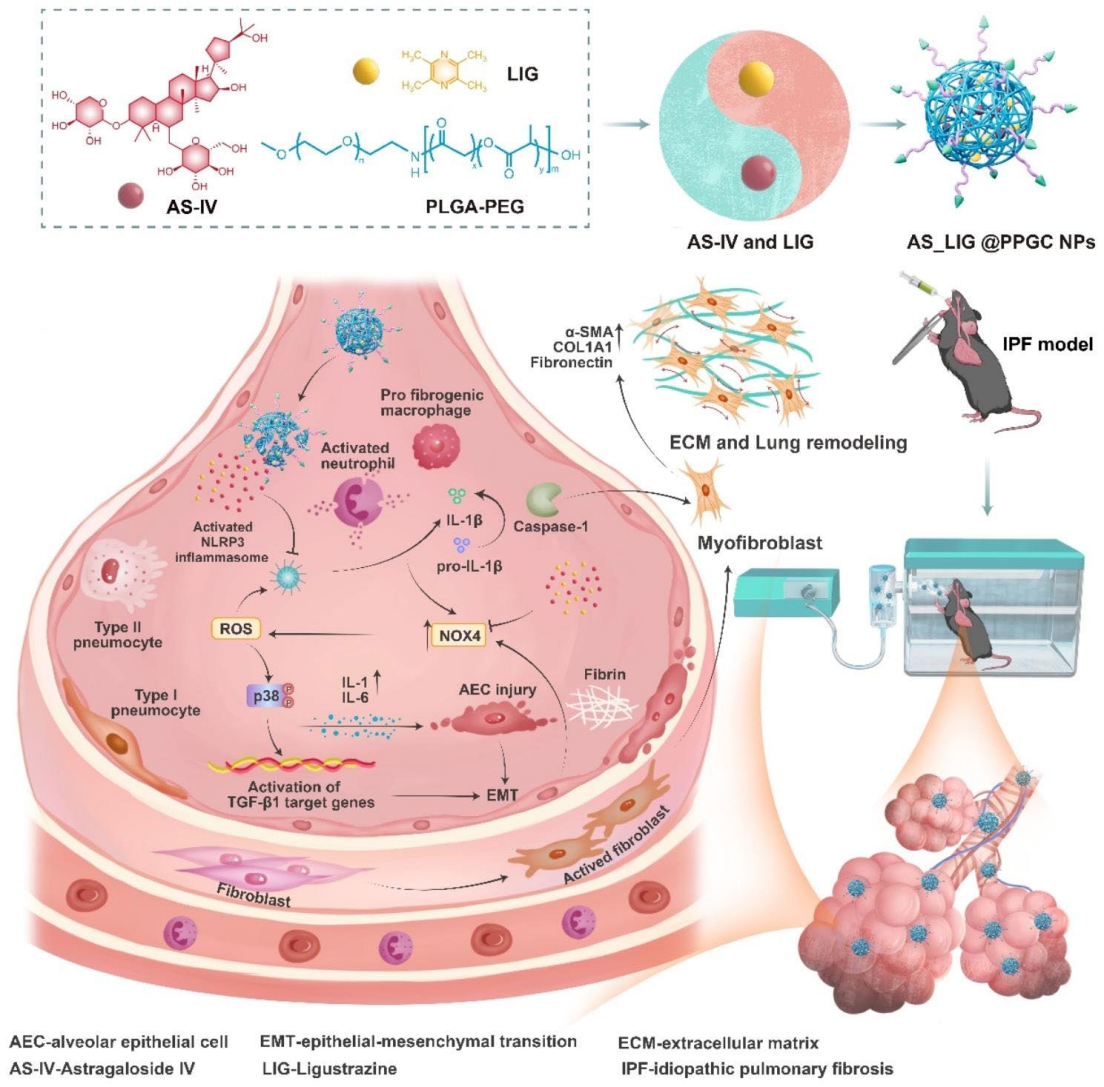
Schematic illustration of AS/LIG/AS_LIG@PPGC NPs for the treatment of lung injury and fibrosis

Based on the antifibrotic effects of AS-IV and LIG, the PEG-PLGA NPs (PPGC NPs) system containing AS-IV and LIG was generated for IPF inhalation therapy for the first time. To achieve this, we prepared PPGC NPs and used drug loading technology to encapsulate AS-IV and LIG. We evaluated the physicochemical properties, drug loading rate, and drug release characteristics of AS_LIG@PPGC NPs and assessed their therapeutic efficacy on IPF through in vivo and in vitro experiments. Finally, we examined the use of AS_LIG@PPGC NPs inhalation therapy for IPF treatment by examining the NADPH oxidase 4 (NOX4)-reactive oxygen species (ROS)-p38 MAPK and NOX4-NLRP3 inflammatory and oxidative stress pathways, as described in Fig. 1. The results of this study will provide a powerful inhaled drug for IPF treatment and will benefit the prognosis of IPF. In addition, this study demonstrated a progressive strategy for the use of compound Chinese medicine and small molecule combinations, as well as the combination of time-honored traditional drugs and advanced nanomedicines. This novel research will improve the application of TCM in the future.

## Materials and methods

### Materials and reagents

Dulbecco’s modified Eagle’s medium (DMEM) and fetal bovine serum (FBS) were purchased from Gibco. TGF-β1 was purchased from Beijing T&L Biotechnology Co., Ltd. Bleomycin sulfate (BLM) was purchased from Shanghai Yuanye Biotechnology Co., Ltd. Cell Counting Kit-8 (CCK-8), and phosphate buffer saline (PBS) were purchased from Beijing Solarbio Science & Technology Co. Ltd. The mouse interleukin-1β (IL-1β), mouse interleukin-6 (IL-6), mouse tumor necrosis factor α (TNF-α), and mouse transforming growth factor β1 (TGF-β1) enzyme-linked immunosorbent assay (ELISA) kits were supplied by Jiangsu Einian Industrial Co. Ltd.

### Antibodies and cells

The murine fibroblast line L-929 were purchased from Xiehe Hospital. The antibodies used in this study were as follows: anti-α-SMA (1:1000 for western blotting, 1:50,000 for immunohistochemistry [IHC], 1:50 for immunofluorescence [IF] ab240654, Abcam, UK), anti-COL1A1 (1:1000 for western blotting, 1:100 for IHC, 1:2,000 for IF, ab270993, Abcam, UK), anti-mouse fibronectin (FN) (1:1000 for western blotting, ab268020, 1:50 for IF Abcam, UK), anti-NOX4 (1:2000 for western blotting, 1:500 for IHC, 14347-1-AP, Proteintech, USA), anti-NLRP3 (1:2000 for western blotting, 1:200 for IHC, 68102-1-Ig, Proteintech, USA), anti-p38 (1:500 for western blotting, 1:1000 for IHC, WLH3870, Wanleibio, China), anti-glyceraldehyde 3-phosphate dehydrogenase (GAPDH) (1:1000 for western blotting, ab8245, Abcam, UK), anti-beta-actin (β-actin) (1:1000 for western blotting, ab8226, Abcam, UK), anti-caspase-1 (1:500 for western blotting, GB11383, Servicebio, China), anti-ASC (1:1000 for western blotting, ab309497, Abcam, UK), anti-IL-1β (1:1000 for western blotting, ab254360, Abcam, UK), anti-mouse IL-18 (1:2000 for western blotting, GB114098, Servicebio, China), and anti-phospho-p38 (p-p38) (1:1000 for western blotting, 28796-1-AP, Proteintech, USA).

### Preparation and characterization of AS_LIG@PPGC NPs

AS-IV, LIG and PLGA-PEG were dissolved in DMSO (1 mL), and then ultrapure water (9 mL) was quickly added to the homogeneous mixture. After being sonicated for 10 min to yield NPs, the NPs were purified by dialysis (molecular weight cutoff 30 kDa) at room temperature for further use. The sizes and morphologies of NPs, AS@PPGC NPs, LIG@PPGC NPs, and AS_LIG@PPGC NPs were observed using transmission electron microscope (TEM, HT7800, Hitachi, Japan). Size measurements of the different formulations were carried out using a Malvern ZEN 3600 Zetasizer instrument. The size measurements of AS_LIG@PPGC NPs were performed by a Malvern ZEN 3600 Zetasizer instrument after 5 days of incubation in PBS.

To determine the encapsulation efficiency (EE) of AS-IV and LIG in PPGC NPs, AS-IV was quantified using high-performance liquid chromatography (lc-2020, Shimadzu, Japan), while LIG was measured using ultraviolet spectrophotometry (Shimadzu, Japan). The formulas for calculating EE and LE are as follows:

$${\rm{EE}}\,\left( {\rm{\% }} \right)\,{\rm{ = }}\,{{{{\rm{W}}_{\rm{0}}}} \mathord{\left/{\vphantom {{{{\rm{W}}_{\rm{0}}}} {{{\rm{W}}_{\rm{1}}}\,{\rm{ \times }}\,{\rm{100}}\,}}} \right.\kern-\nulldelimiterspace} {{{\rm{W}}_{\rm{1}}}\,{\rm{ \times }}\,{\rm{100}}\,}}{\rm{\% }}$$

W_0_ and W_1_ represent the weight of the loaded AS-IV and LIG in the NPs and the total weight of AS-IV and LIG added, respectively, while W represents the total weight of the NPs.

### Cellular uptake and cell viability assay

To measure uptake efficiency, L-929 cells were seeded in 24-well plates at a density of 3 × 10^4^ per well. After 6 h of incubation, 100 µg/mL TGF-β1 was added to the L-929 cells. After another 24 h, the cells were treated with different concentrations of 1,1’-dioctadecyl-3,3,3’,3’-tetramethylindodicarbocyanine,4-chlorobenzenesulfonate salt ([DiD], DiD@PPGC NPs). The uptake efficiencies in the different groups were analyzed by flow cytometry (BD, FACSAriall) and imaged by inverted fluorescence microscopy. We employed a standard CCK-8 assay, utilizing the Multiskan FC microplate reader (ThermoFisher Scientific, 1,410,101), to assess the cell viability of L-929 cells under various concentration treatments. The assay relied on measuring the alterations in absorbance at 450 nm, providing a reliable indication of cellular metabolic activity. Cell viability rate (%) = (absorbance of experimental group − absorbance of blank well) / (absorbance of control group − absorbance of blank well) × 100%.

### In vitro ROS analysis

L-929 cells were seeded on 6-well plates and incubated overnight. First, the cells were activated with TGF-β1, and then, different treatment groups were incubated with L-929 cells for 24 h. Next, the ROS probe 2’,7’-dichlorodihydrofluorescein diacetate (DCFH-DA) was added to the dishes under 5% CO_2_ and incubated in the dark for 15 min at 37 °C. Finally, we employed confocal laser scanning microscopy (CLSM, Nikon N-SIM, Japan) to assess intracellular ROS (λ excitation/λ emission = 488 nm/525 nm).

### Immunofluorescence assay

Briefly, L-929 cells were seeded in 24-well plates at a density of 3 × 10^4^ per well. After 6 h of incubation, 100 µg/mL TGF-β1 was added to the wells. Then, the cells were treated with the different formulations (naked NPs, AS@PPGC NPs, LIG@PPGC NPs, and AS_LIG@PPGC NPs) for another 24 h. Finally, the cells were stained with antibodies (α-SMA, COL1A1, FN), and immunofluorescence images were recorded by CLSM (Nikon N-SIM, Japan).

### Animals

C57BL/6J mice (18–20 g, 6–8 weeks, male) were purchased from Beijing Vital River Laboratory Animal Technology Co. Ltd. The mice were randomly divided into six groups: control, model, naked NPs, AS@PPGC NPs (25 mg/kg), LIG@PPGC NPs (50 mg/kg), and AS_LIG@PPGC NPs (AS-IV: 25 mg/kg, LIG: 50 mg/kg). Except for those in the control group, the mice were intratracheally injected with BLM sulfate at a dose of 1.5 U/kg to establish experimental lung injury and pulmonary fibrosis models. The treatments were administered via inhalation on Days 3, 6, 10, 13, 16, and 19 after BLM insult using an air compressor nebulizer. Then, the treated mice were sacrificed on Day 8 and Day 22. All animal experiments were performed in accordance with the China Public Health Service Guide for the Care and Use of Laboratory Animals. Experiments involving mice and the protocols were approved by the Institutional Animal Care and Use Committee of Tsinghua University (AP#15-LRT1).

### Tissue distribution of AS_LIG@PPGC NPs

The mice were first exposed to 1,1’-dioctadecyl-3,3,3’,3’-tetramethylindotricarbocyanine iodide ([DiR], DiR@PPGC NPs) via inhalation for 30 min. Subsequently, the lung, heart, liver, and kidney were examined at different time points (0, 2, 4, 8, 12, and 24 h) using an in vivo fluorescence imaging system (PerkinElmer, USA). The lung tissues were then collected, rapidly frozen, and embedded in optimum cutting temperature (OCT) medium (Sakura, Japan) on dry ice. Histological cross-sections (10 μm thick) of lung tissues were prepared and stained with 4’,6-diamidino-2-phenylindole (DAPI) in PBS for 2 min to visualize the nuclei. Finally, the sections were washed three times with PBS and imaged using a Vectra Polaris™ automated quantitative pathology imaging system (PerkinElmer, USA), which enables accurate and reproducible analysis of tissue samples.

### Determination of proinflammatory and profibrotic cytokines

L-929 cells were seeded in 24-well plates at a density of 3 × 10^4^ per well. After 6 h of incubation, 100 µg/mL TGF-β1 was added to each well. Then, the cells were treated with different formulations of NPs, AS@PPGC NPs, LIG@PPGC NPs, and AS_LIG@PPGC NPs for another 24 h. the expression levels of the inflammatory cytokines IL-1β, IL-6, TNF-α, and TGF-β1 in each group were measured by ELISA.

To collect bronchoalveolar lavage fluid (BALF), the trachea was exposed, and a tracheotomy was performed to introduce a 24G needle. A 1 mL syringe with 800 µL of cold sterile PBS was used to gently wash the lungs three times through the trachea, and the recovery rate was approximately 80%. Once collected, BALF samples were centrifuged at 400 × g for 10 min at 4 °C and stored at -80 °C until use. Subsequently, the expression levels of IL-1β, IL-6, TNF-α, and TGF-β1 in the BALF samples were measured by ELISA.

### Histological analysis

The left lungs in the different groups were quickly immersed in 4% paraformaldehyde (PFA) for 24 h at room temperature, approximately 20–25 °C, Half of the lung tissues were dehydrated and embedded in paraffin, and 4 μm sections were prepared and stained with hematoxylin and eosin (H&E), Masson’s trichrome, and picrosirius red. Histologic sections of the heart, kidney, liver, spleen, and lung were used for H&E staining.

### Immunohistochemistry

The remaining lung tissue was fixed in 4% PFA at 4 °C for 1 h. After sucrose gradient dehydration, the tissue was embedded in OCT compound. Subsequently, 10 μm cryosections were prepared for immunohistochemical and immunofluorescence analysis.

For immunohistochemical analysis of α-SMA, COL1A1, NOX4, NLRP3 and p-p38, tissue sections were treated with appropriate primary antibodies followed by incubation with horseradish peroxidase (HRP)-conjugated secondary antibodies. The substrate 3,3’-diaminobenzidine (DAB) was used for direct visualization of the target proteins.

### Western blotting

The samples were subjected to sodium dodecyl sulfate-polyacrylamide gel electrophoresis (SDS-PAGE) separation at an equal protein mass (10 µg per well) and subsequently transferred onto polyvinylidene difluoride (PVDF) membranes. To minimize non-specific binding, the membranes were blocked with 5% bovine serum albumin (BSA) at room temperature (40 rpm/min) for 1 h. Subsequently, the membranes were incubated overnight at 4 °C with the appropriate primary antibodies, followed by three washes with a solution containing tris-buffered saline with Tween 20 (TBST) and low-speed shaking on a rocker (80 rpm/min) for 10 min each. After that, the membranes were incubated with suitable secondary antibodies at room temperature for approximately 2 h. The membranes were then washed three times with a TBST solution (80 rpm/min). Finally, an electrochemiluminescence chromogenic substrate was added, and the target bands were visualized using the Tanon 5200 Multi fully automated chemiluminescence/fluorescence image analysis system (Guangzhou, China). The chemiluminescence signals obtained were analyzed using ImageJ software (NIH, USA).

### Molecular docking

Protein‒ligand docking analysis was performed to predict the interactions and binding modes between NOX4, NLRP3 and AS_IV/LIG. The PDB files of the 3D structures of NOX4 (PDB ID: 6T1U) and NLRP3 (PDB ID: 8ERT) were downloaded from the RCSB PDB database and imported into PyMOL software to remove solvent molecules and ligands. The SDF files of AS-IV (PubChem CID: 13,943,297) and LIG (PubChem CID:14,296) were downloaded from the PubChem database and converted to the mol2 format using OpenBabel software. The ligands and proteins were then separately imported into Autodock, a molecular docking program based on the Lamarckian genetic algorithm, to search for all possible binding models in the translation and rotation space between the proteins and ligands. Based on the maximum scoring function, the optimal binding mode of the protein‒ligand complex was selected, and the output was the protein‒ligand complex. The amino acid residues and hydrogen bonding forces of the protein‒ligand binding was predicted using PyMOL software.

### Transcriptomic analysis

We euthanized mice in the model and AS_LIG@PPGC NP groups on Day 22 and extracted left lung tissues from three mice in each group. The lung tissues were immediately placed in cryotubes, frozen in liquid nitrogen, and then sent to Wuhan Bio-Raid Biotechnology Co., Ltd. (Wuhan, China) for RNA sequencing (RNA-seq) analysis. RNA-seq involves sample processing, RNA extraction, library preparation, and high-throughput sequencing using the Illumina HiSeq Xten/NovaSeq 6000/T7 platforms. Basic bioinformatics analysis included differential gene expression analysis (FDR < 0.05 & |log2FC|≧1), GO classification analysis of differentially expressed genes, KEGG pathway visualization, and GSEA.

### Statistical analysis

The results were analyzed using GraphPad Prism software (version 9.5.1, USA) and are presented as the means ± standard deviation (SD). One-way analysis of variance (ANOVA) was used for statistical analyses during data evaluation. After performing ANOVA, we performed post hoc multiple comparison analyses using the Student-Newman‒Keuls (SNK) method to determine which groups had significant differences. This analysis was based on a multiple comparison correction method and was considered significant when the *P* value was less than 0.05.

## Results

### Preparation and characterization of AS_LIG@PPGC NPs

**Fig. 2:**
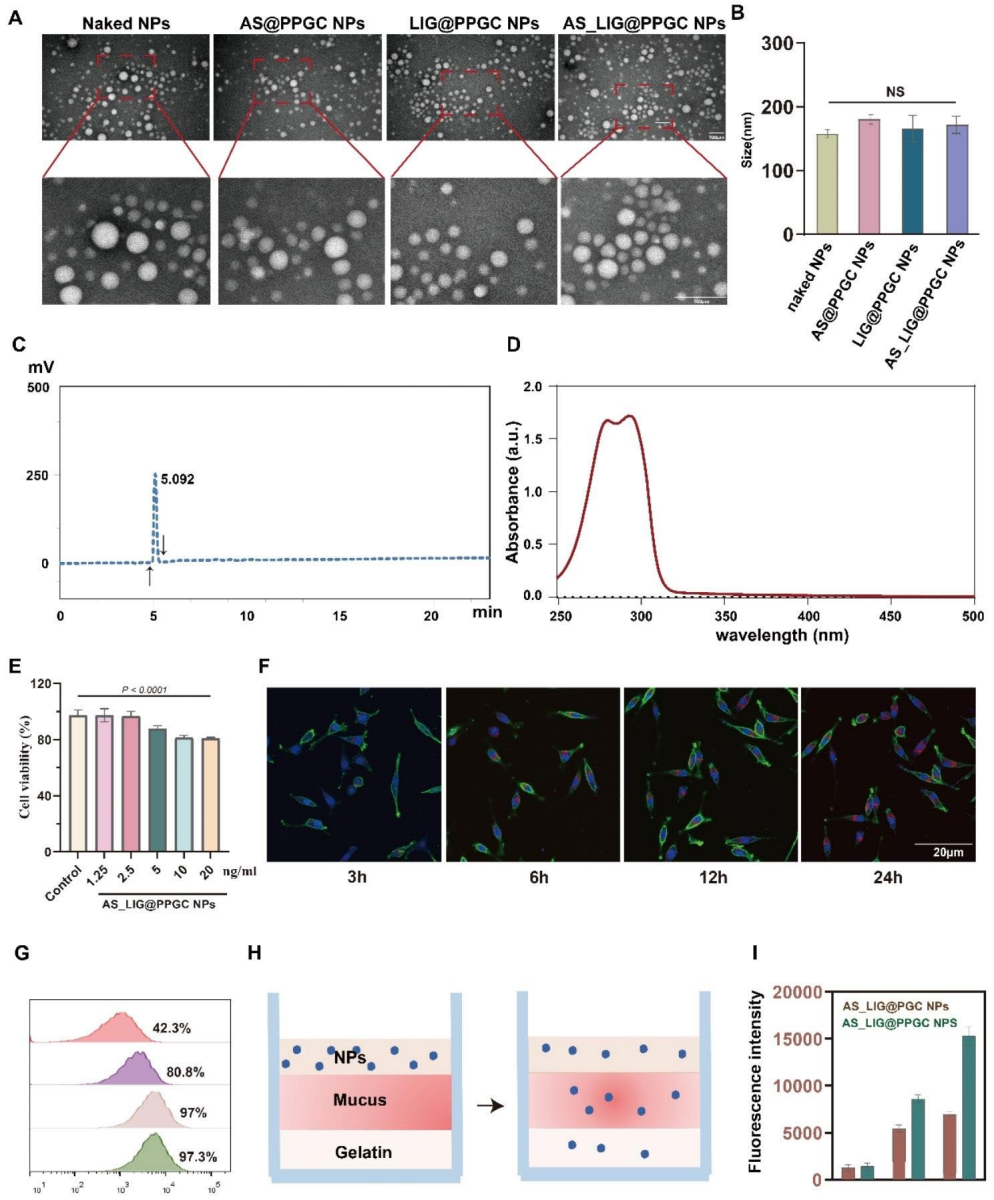
The characteristic of AS_LIG@PPGC NPs. (**A**). The TEM image of naked NPs, AS@PPGC NPs, LIG@PPGC NPs, and AS_LIG@PPGC NPs. Scale bar = 100 μm. (**B**). The sizes of naked NPs, AS@PPGC NPs, LIG@PPGC NPs, and AS_LIG@PPGC NPs mearsured by DLS.(n = 3) (**C** to **D**). The encapsulation efficiencies of AS and LIG in AS_LIG@PPGC NPs. (**E**). Cell viabilities of of TGF-β1 treated L-929 cells incubated with AS_LIG@PPGC NPs different concentrations. (**F**). CLSM images of time-dependent cellular uptake of AS_LIG@PPGC NPs in TGF-β treated L-929 cells, Scale bar = 20 μm. The results were expressed as the mean ± SD (n = 3). (**G**). Flow cytometry analysis of uptake efficiencies in TGF-β1 treated L-929 cells. (**H** to **I**). Penetration of NPs with and without PEG coating in an artificial mucus model. The results were expressed as the mean ± SD (n = 3)

PLGA NPs encapsulated with AS-IV and LIG were prepared by nanoprecipitation sonication. The naked NPs, AS@PPGC NPs, LIG@PPGC NPs, and AS_LIG@PPGC NPs remained spherical morphology as observed by TEM, with sizes of 50 nm in their dry state (Fig. 2A). The products exhibited hydrated diameters of approximately 200 nm, as determined by DLS measurements (Fig. 2B). The size stability of AS_LIG@PPGC NPs was maintained for 5 days (Figure S1). Meanwhile, no changes of AS_LIG@PPGC NPs size were observed after inhalation (Figure S2). The contents of AS-IV and LIG in the as-prepared NPs were measured by UV and HPLC, respectively. Figure 2C and D show that the encapsulation efficiencies of AS-IV and LIG in the NPs were 37% and 74.1%, respectively. These results indicated that AS-IV and LIG were successfully loaded. Fibroblasts are the primary targets in IPF. Therefore, to assess AS_LIG@PPGC NPs uptake by host cells, AS_LIG@PPGC NPs were first labeled with DiD and then co-incubated with L-929 mouse fibroblasts. The CCK-8 results demonstrated that the viability of L-929 cells was still greater than 80% when the concentration of AS_LIG@PPGC NPs reached 20 ng/mL, and there was no apparent cytotoxicity (Fig. 2E). The fluorescence signal intensity showed time-dependent uptake of AS_LIG@ PPGC NPs by the cells (Fig. 2F, G). Furthermore, different concentrations of DiD-labeled AS_LIG@ PPGC NPs were added to L-929 cells and incubated for 24 h to evaluate cell viability. In addition, an artificial mucus model was used to investigate the impact of PEG coating on the transmucosal penetration of DiD-labeled NPs (Fig. 2H). The resulting NPs (with or without PEG decoration) were added to the artificial mucus at equivalent doses of DiD@NPs, and the fluorescence intensity of the agarose gel layer was analyzed after penetration. As shown in Fig. 2I, the fluorescence signal intensities with the PEG decoration were significantly higher than those without the PEG decoration, suggesting that the transmucosal penetration of NPs was significantly enhanced by the PEG coating. In addition, the magnitude of the increase in NPs penetration markedly increased with time.

### Biodistribution of PPGC NPs in the lungs after inhalation

**Fig. 3:**
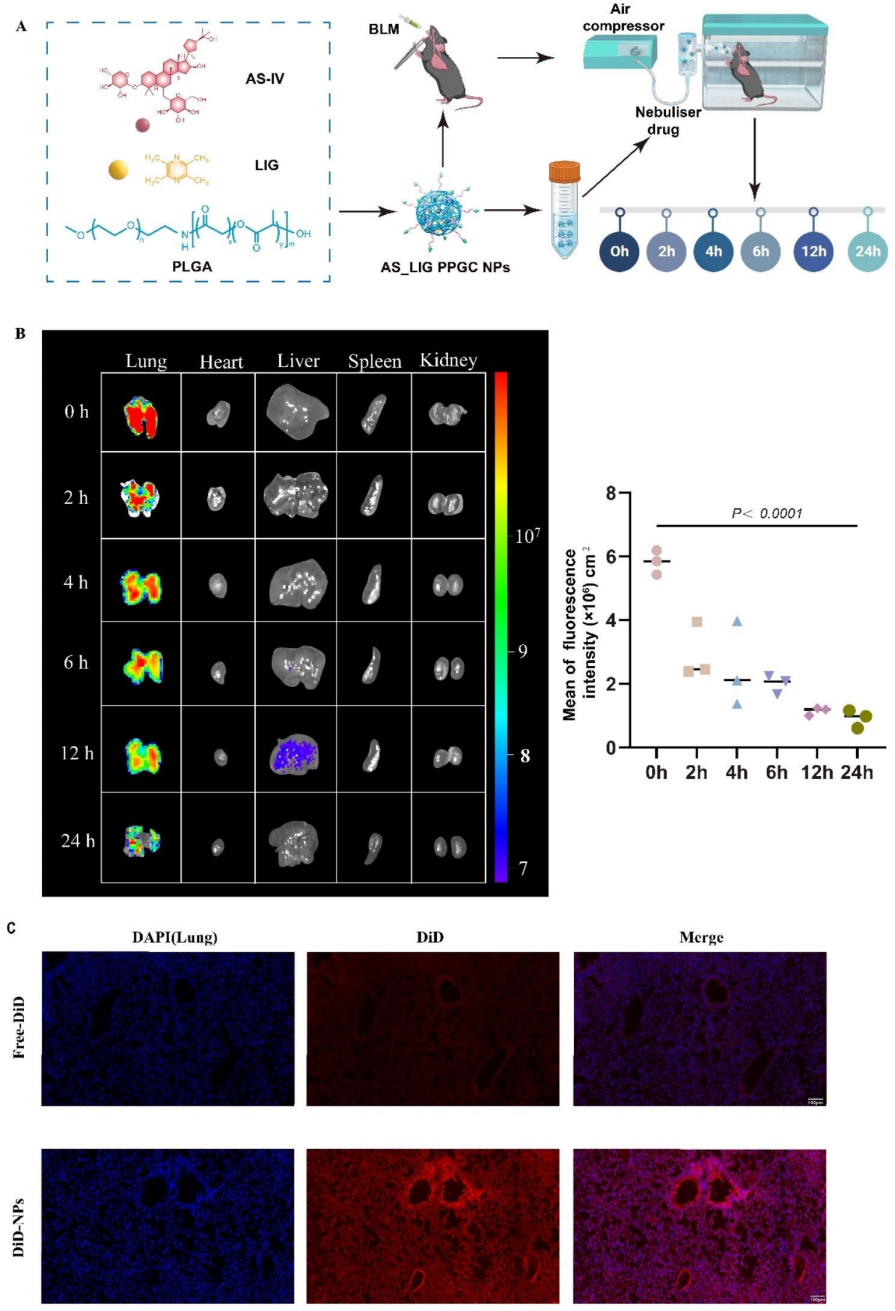
Tissue distribution of AS_LIG@PPGC NPs in mice after inhalation. (**A**). Flowchart of the BLM-induced IPF model at different time points following inhalation of AS_LIG PPGC NPs (n = 18). (**B**). Fluorescence images of lung, heart, liver, spleen, and kidney at predetermined time points (0, 2, 4, 6, 12 and 24 h). (**C**). Fluorescence images of DAPI (blue) and DiD (red) distribution in lung tissue sections after inhalation of AS_LIG@PPGC NPs for 30 min. Scale bar = 100 μm

To evaluate the pulmonary targeting ability of PPGC NPs delivered via compressed air nebulization, we labeled PPGC NPs with DiR and used an IVIS spectrum imaging system to study tissue distribution at 0, 2, 4, 6, 12, and 24 h after inhalation (Fig. 3A). Our results demonstrated that after the inhalation of DiR-labeled PPGC NPs, strong fluorescence signals were detected in lung tissue, indicating the excellent pulmonary targeting of the NPs. The intensity of the fluorescence signal in the lung gradually decreased with time but remained strong at 12 h, suggesting that the inhaled PPGC NPs exhibited persistent stability. At 24 h, the fluorescence signal in lung tissue gradually weakened, possibly due to the metabolism of the NPs (Fig. 3B). Therefore, these findings suggest that compressed air nebulization is a feasible targeted pulmonary drug delivery method, and PPGC NPs can be efficiently enriched in the lungs by inhalation.

To further access the lung-targeting of PPGC NPs after nebulization, we subsequently collected lung tissues from mice following the inhalation of DiD-labeled NPs and performed immunofluorescence imaging (Fig. 3C). The results showed that the fluorescence signal of PPGC NPs after nebulization was stronger than that of free-DiD. To deeply investigate the specific distribution of NPs in the lungs following inhalation, we performed IF analysis on different lung lobes (right upper lobe, middle lobe, lower lobe, and left lung lobe) at different time points. As shown in Figure S5, the NPs exhibited a widespread distribution throughout the entire lung lobes after inhalation, and they persisted in the lungs for more than 24 h. Consistent with our predictions, the retention of NPs in the lungs decreased over time, with a predominant deposition observed in the right lower lobe and left lung. These findings collectively demonstrate that the observed deposition pattern aligns with the physiological structure of the lungs. All these results indicated that the PPGC NPs can effectively target lung tissues.

### Evaluation of the antifibrotic and anti-inflammatory effects of AS_LIG@PPGC NPs in vitro

**Fig. 4:**
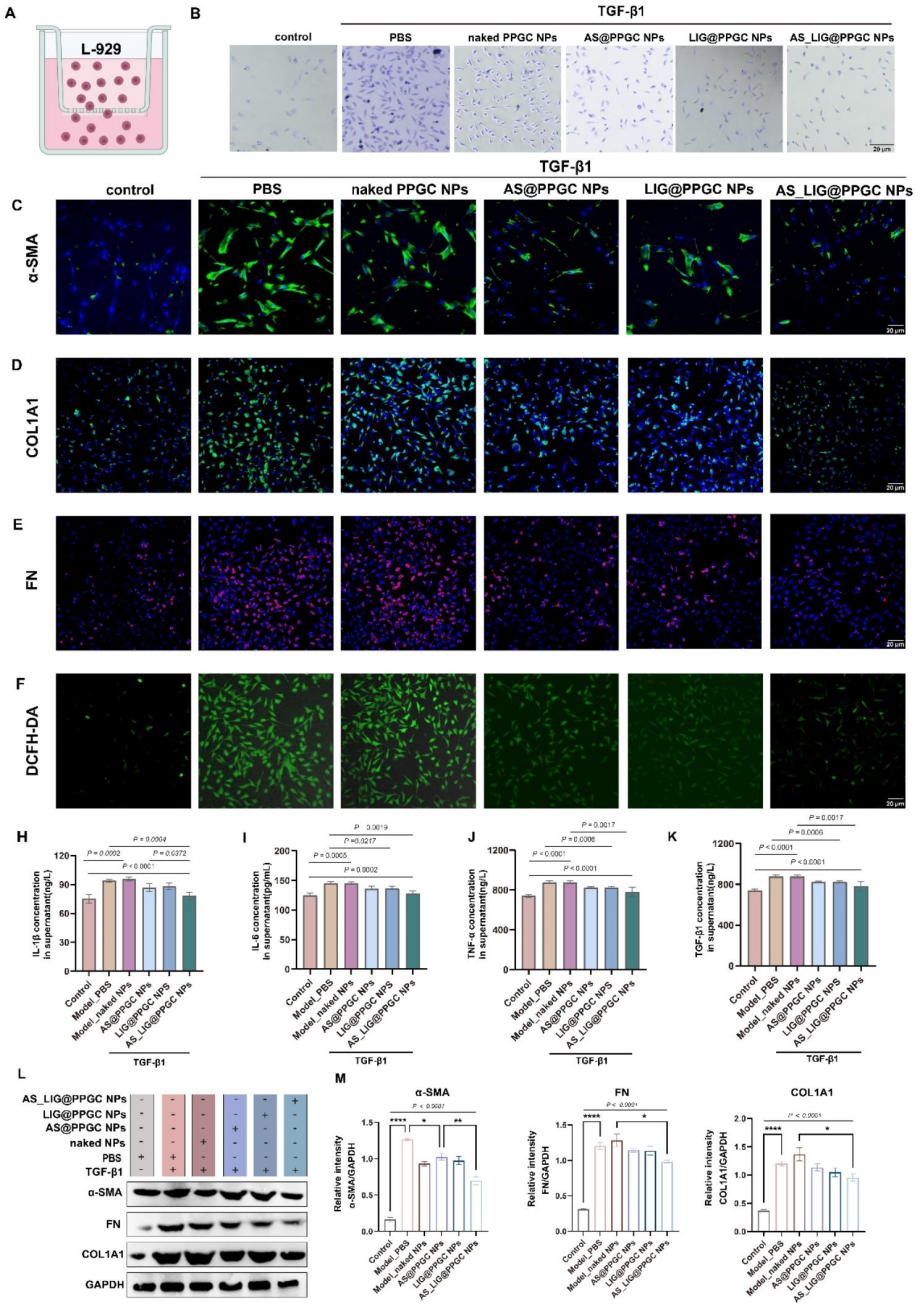
AS/LIG/AS_LIG@PPGC NPs reduce the secretion of pro-inflammatory and profibrotic cytokines and thus inhibit the activation of myofibroblasts in vitro. (**A**). Schematic illustration of transwell migration assay. (**B**). Images of stained cells migrating from the upper chamber to the lower chamber with different treatments Scale bar = 20 μm. (**C** to **E**). Representative immunofluorescence images of α-SMA, COL1A1, and FN after different treatments. Scale bar = 20 μm. (**F**). Images of of DCFH-DA lablled ROS in TGF-β1 treated L-929 cells after different treatments. Scale bar = 20 μm. (**E** to **K**). Expression levels of typical pro-inflammatory and profibrotic cytokines (TNF-α, IL-6, IL-1β, and TGF-β1) secreted by TGF-β1 treated L-929 cells after different treatments. The results were expressed as the mean ± SD (n = 3). (**L**, **M**). Western blotting analysis of expression and quantification of proteins related to pulmonary fibrosis (α-SMA, COL1A1, FN) in the cells treated as indicated. * *P* < 0.05, **P < 0.01, ***P < 0.001, and ****P < 0.0001. n.s., not significant, *P* > 0.05

With the development of pulmonary fibrosis, the migration of fibroblasts and myofibroblasts into fibroblastic foci is a critical event. We evaluated the impact of AS_LIG@PPGC NPs on the migration of fibroblasts with a transwell migration assay (Fig. 4A). As shown in Fig. 4B, the migration efficiency of L-929 cells treated with TGF-β1 was determined. The results indicated that migration of L-929 cells could be hampered by intervention with AS@PPGC NPs, LIG@PPGC NPs, and AS_LIG@PPGC NPs. And AS_LIG@PPGC NPs exhibited the most significant inhibitory effect. Then, we further evaluated the antifibrotic effects of the different treatments on TGF-β1-pretreated L-929 cells. The immunofluorescence staining results showed that the intensities of typical protein markers (α-SMA, COL1A1, and FN) were significantly reduced by AS_LIG@PPGC NPs treatment (Fig. 4C-E). However, AS@PPGC NPs and LIG@PPGC NPs showed a less pronounced effect than AS_LIG@PPGC NPs. To investigate the anti-inflammatory and antifibrotic effects of AS_LIG@ PPGC NPs, TGF-β1-pretreated L-929 cells were first incubated with the different treatments for 24 h. The supernatants were collected for ELISA. The cells with different treatments were stained with DCFH-DA probe for CLSM observation. The fluorescence intensity of DCFH-DA probe was significantly reduced by treatment with AS_LIG@PPGC NPs, demonstrating the efficient antioxidant effects of AS_LIG@PPGC NPs (Fig. 4F). Interestingly, the levels of proinflammatory cytokines (IL-6, TNF-α, IL-1β) and profibrotic cytokines (TGF-β1) were significantly reduced by AS_LIG@PPGC NPs treatment, but AS@PPGC NPs or LIG@PPGC NPs alone hardly eliminated these factors in TGF-β1-pretreated L-929 cells (Fig. 4H-K). The levels of IL-6 in the model group and naked NPs group were increased by 1.16-fold and 1.15-fold, respectively, compared to those in the normal group. However, the levels of IL-6 were reduced to 112.71 pg/ml and 112.85 pg/ml after AS@PPGC NPs and LIG@PPGC NPs treatments while AS_LIG@PPGC NPs led to a reduction of approximately 13%. Similar changes were observed in TNF-α, IL-1β, and TGF-β1.

Finally, we performed western blotting analysis to investigate the expression levels of typical fibrosis-associated proteins, such as α-SMA, COL1A1, and FN, in vitro. The results demonstrated that treatment with AS_LIG@PPGC NPs significantly reduced the expression levels of these proteins compared to those in the model group (Fig. 4L, M). These findings suggested that AS_LIG@ PPGC NPs may exert notable antifibrotic effects through anti-inflammatory and antioxidant pathways.

### Inhalation of AS/LIG/AS_LIG@PPGC NPs mitigates BLM-induced pulmonary fibrosis

**Fig. 5:**
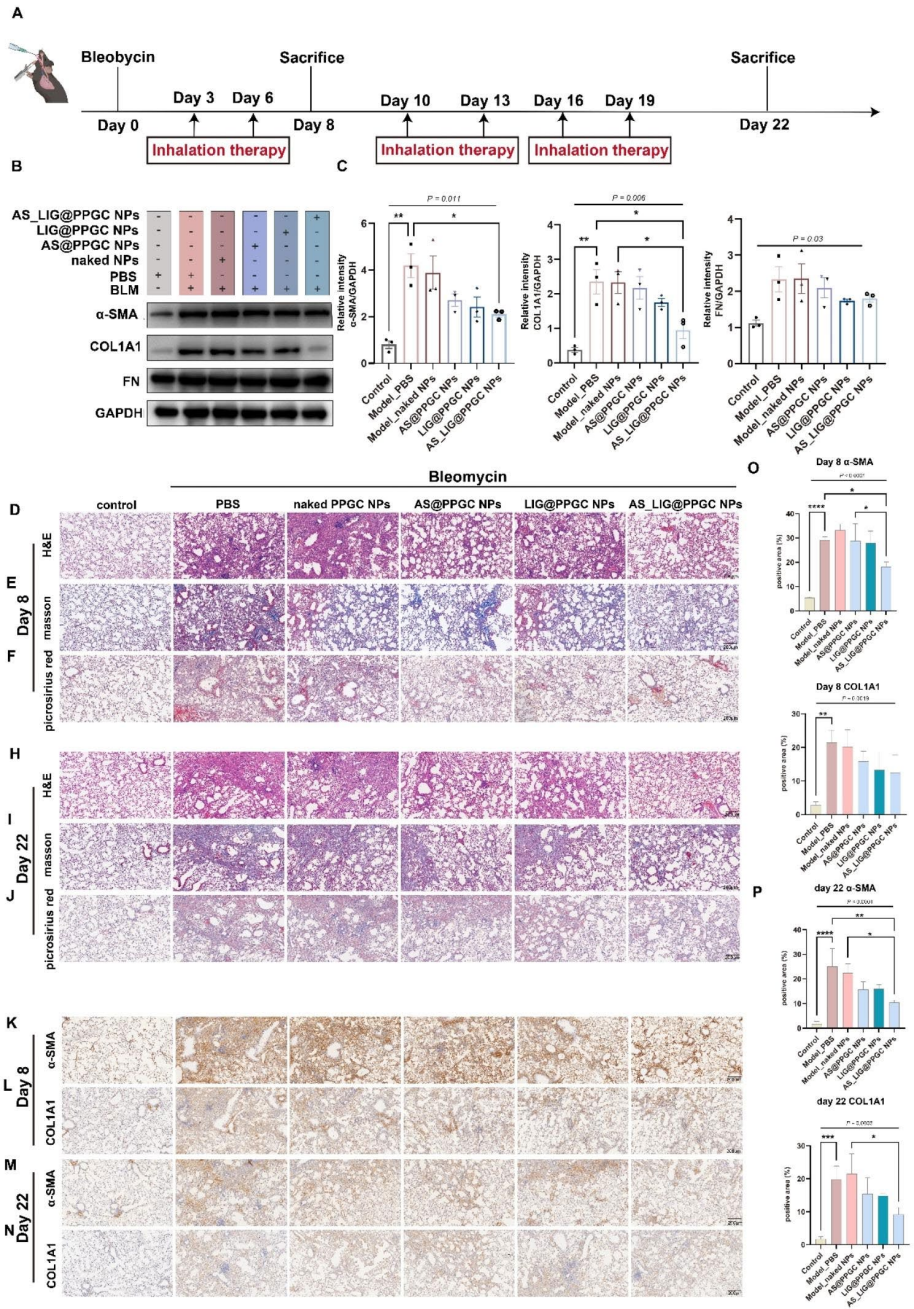
AS/LIG/AS_LIG@PPGC NPs inhalation reverses lung fibrosis caused by BLM. (**A**). The schematic of the study design; n = 8 biological independent animals per group. (**B**, **C**). Western blotting analysis of expression and quantification of proteins related to pulmonary fibrosis (α-SMA, COL1A1, FN) in the lung tissue treated as indicated. Data were represented as mean ± SD (n = 3). (**D** to **F**). Histological analysis of lung sections on day 8. (**D**). representative H&E staining. (**E**). Masson’s trichrome staining [muscle fibers and erythrocytes (red), collagen (blue), and nuclei (black-purple)]. (**F**). Picrosirius red staining (collagen types I and III) (red). Scale bar = 200 μm. (**H** to **J**). Histological analysis of lung sections on day 22. (**H**). representative H&E staining. (**I**). Masson’s trichrome staining [muscle fibers and erythrocytes (red), collagen (blue), and nuclei (black-purple)]. (**J**). Picrosirius red staining (collagen types I and III) (red). Scale bar = 200 μm. (**K**, **L**, **O**). IHC staining analysis of lung sections and quantification of positive area of proteins related to pulmonary fibrosis on day 8. Scale bar = 200 μm. (**K**). IHC staining analysis of lung sections of α-SMA. (**L**). IHC staining analysis of lung sections of COL1A1. (**O**). quantification of positive area about α-SMA, COL1A1. (**M**, **N**, **P**). IHC staining analysis of lung sections and quantification of positive area of proteins related to pulmonary fibrosis on day 22. Scale bar = 200 μm. (**M**). IHC staining analysis of lung sections of α-SMA. (**N**). IHC staining analysis of lung sections of COL1A1. (**P**). quantification of positive area of α-SMA, COL1A1. * *P* < 0.05, **P < 0.01, ***P < 0.001, and ****P < 0.0001, n.s., not significant, *P* > 0.05

After confirming the strong anti-inflammatory and antioxidant effects in vitro, the mouse IPF models for in vivo treatment was established (Fig. 5A). Then, we collected lung tissue on Day 8 and Day 22 after BLM induction and evaluated pulmonary fibrosis levels using H&E staining, Masson staining, and picrosirius red staining. H&E staining revealed that the airway structures in the normal group were clear and intact, and there was no inflammatory cell infiltration or bleeding in the alveoli. However, on Day 8, the PBS and naked PPGC NPs groups showed thickening of the airway walls, significant infiltration of inflammatory cells, and bleeding in the alveoli. In contrast, inhalation of AS@PPGC NPs, LIG@PPGC NPs, and AS_LIG@PPGC NPs reduced the infiltration of inflammatory cells and decreased the occurrence of alveolar bleeding (Fig. 5D). On Day 22, the airway structures in the PBS and naked PPGC NP groups were disrupted, and there was significant thickening of the airway walls, widening of the alveolar septa, and inflammatory cell infiltration. However, treatment with AS@PPGC NPs, LIG@PPGC NPs, and AS_LIG@PPGC NPs significantly reduced the severity of BLM-induced fibrosis (Fig. 5H). Masson staining and picrosirius red staining further confirmed a significant reduction in collagen deposition and parenchymal disruption after the inhalation of AS@PPGC NPs and LIG@PPGC NPs (Fig. 5E-J).

Furthermore, we used immunohistochemistry and western blotting to examine fibrotic markers in mouse lung tissue. Immunohistochemical analysis revealed significant reductions in the protein expression of α-SMA and COL1A1, which are typical protein markers of lung fibrosis, following the inhalation of AS@PPGC NPs, LIG@PPGC NPs, and AS_LIG@PPGC NPs (Fig. 5K-P). Consistent with these findings, western blot analysis demonstrated that the inhalation of these NPs effectively reduced the protein expression of α-SMA, a marker of myofibroblasts, as well as the extracellular matrix components COL1A1 and FN (Fig. 5B, C). Semiquantitative analysis of α-SMA expression showed that the levels in the model group and naked PPGC NPs group were increased by approximately 6-fold and 5-fold, respectively Compared to naked PPGC NPs, AS@PPGC NPs, LIG@PPGC NPs, and AS_LIG@PPGC NPs significantly reduced the expression of α-SMA, especially AS_LIG@PPGC NPs, which led to an approximately 88% reduction in in protein expression. Similar changes were observed in COL1A1 and FN expression.

The findings of this study indicated that AS@PPGC NPs, LIG@PPGC NPs, and AS_LIG@PPGC NPs hold potential as therapeutic interventions for IPF, especially for IPF induced by BLM, and they effectively alleviated the pathological manifestations of the disease. Moreover, the combination of AS-IV and LIG showed better therapeutic effects and warrant further investigation as a potential treatment strategy for IPF.

### Inhalation of AS/LIG/AS_LIG@PPGC NPs attenuates NOX4-NLRP3 signaling pathway activation

**Fig. 6:**
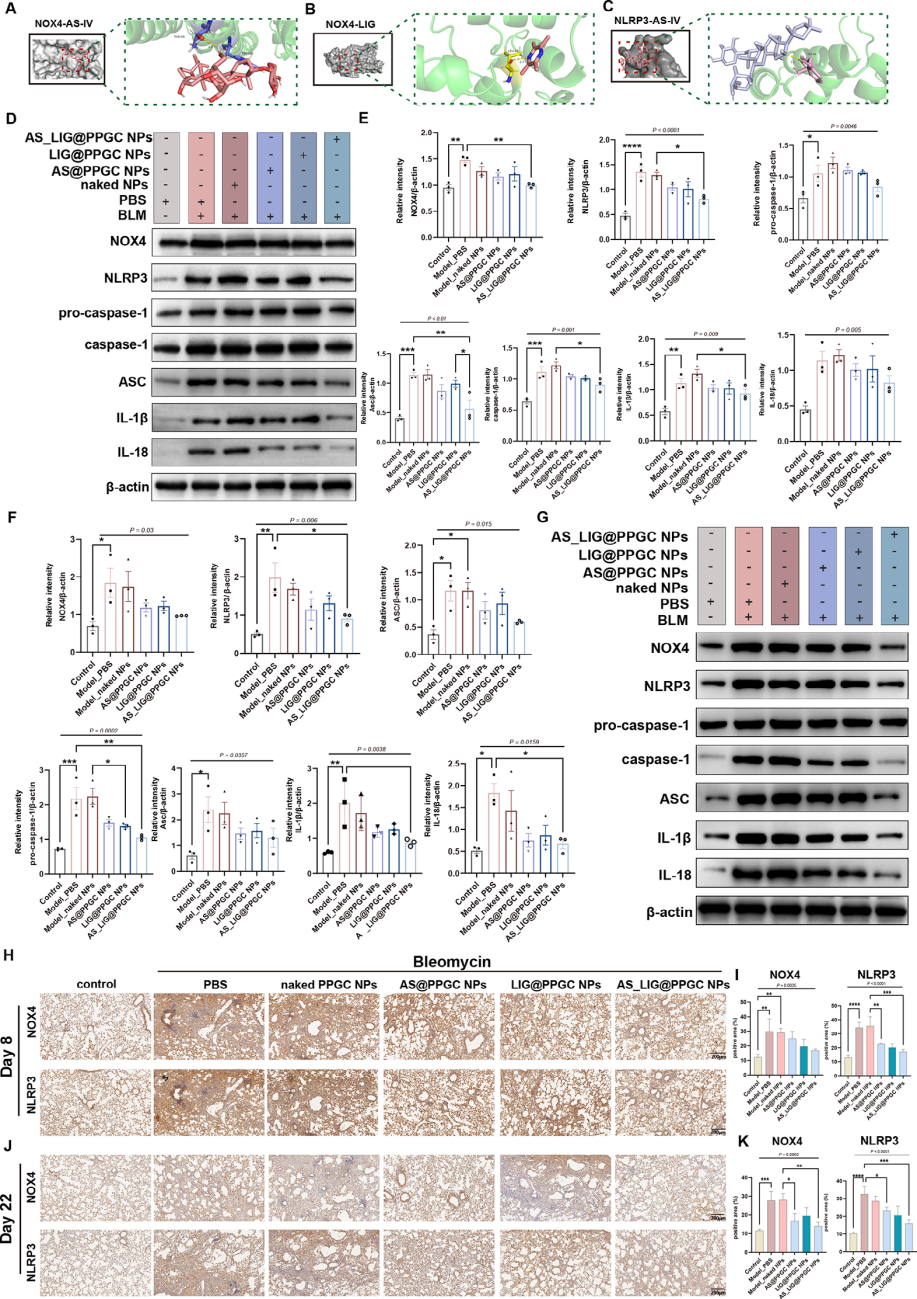
AS/LIG/AS_LIG@PPGC NPs inhalation reverses lung fibrosis caused by BLM through NOX4-NLRP3 signalling pathway. (**A** to **C**). Representative molecular docking of AS-IV and LIG with NOX4 and NLRP3 proteins, respectively. (**A**). Molecular docking of AS-IV with NOX4. (**B**). Molecular docking of LIG with NOX4. (**C**). Molecular docking of AS-IV with NLRP3. (**D**, **E**). Western blotting analysis of expression and quantification of proteins related to NOX4-NLRP3 signalling pathway (NOX4, NLRP3, pro-caspase-1, caspase-1, ASC, IL-1β, and IL-18) in the lung sections on day 22 treated as indicated. (**F**, **G**). Western blotting analysis of expression and quantification of proteins related to NOX4-NLRP3 signalling pathway (NOX4, NLRP3, pro-caspase-1, caspase-1, ASC, IL-1β, and IL-18) in the cells treated as indicated. (**H**, **I**). IHC staining analysis of lung sections and quantification of proteins related to NOX4-NLRP3 signalling pathway on day 8. Scale bar = 200 μm. (**H**). IHC staining analysis of lung sections of NOX4, NLRP3. (**I**). Quantification of proteins of NOX4, NLRP3. (**J**, **K**). IHC staining analysis of lung sections and quantification of positive area of proteins related to NOX4-NLRP3 signalling pathway on day 22. Scale bar = 200 μm. (**J**). IHC staining analysis of lung sections of NOX4, NLRP3. (**K**). Quantification of positive area of NOX4, NLRP3. * *P* < 0.05, **P < 0.01, ***P < 0.001, and ****P < 0.0001, n.s., not significant, *P* > 0.05

After confirming their excellent therapeutic effects in vivo, we further performed automatic docking calculations for AS-IV and LIG using the Autodock tool and focused on their binding affinities with NOX4 and NLRP3. Our results demonstrated that AS-IV and LIG could successfully bind to NOX4, while AS-IV could also stably bind to NLRP3. To gain a clearer understanding of the molecular interactions, we further used PyMOL software to visualize the hydrogen bonds and docking pockets (Fig. 6A-C). Based on these findings, we hypothesized that AS-IV and LIG could exert antifibrotic effects by inhibiting the NOX4-NLRP3 signaling pathway.

In the following experiments, we used in vitro, and in vivo methods combined with western blotting and IHC experiments to further validate the molecular docking results. The western blotting results showed that the NOX4-NLRP3 signaling pathway was significantly activated in the BLM-induced IPF mouse model. Specifically, in the lungs of mice with the BLM-induced IPF model, there was a significant upregulation of NOX4 protein expression. This upregulation leaded to the catalysis of a substantial production of ROS, subsequently activating the NLRP3 inflammasome. After inhalation of AS@PPGC NP, LIG@PPGC NP, and AS_LIG@PPGC NPs, the protein levels of NOX4 and NLRP3 in lung tissue in the model group mice, as well as the protein levels of downstream ASC, pro-caspase-1, and caspase-1 in the NOX4-NLRP3 signaling pathway, were significantly increased. Additionally, the protein levels of IL-1β and IL-18, which are processed by caspase-1, were significantly increased. In the AS@PPGC NP, LIG@PPGC NP, and AS_LIG@PPGC NP groups, the most significant inhibitory effect was observed in the AS_LIG@PPGC NPs group (Fig. 6D, E). Furthermore, we obtained similar results in vitro (Fig. 6F, G).

In addition, we used IHC to measure the protein levels of NOX4 and NLRP3 in mouse lung tissue on Days 8 and 22, and the staining results were consistent with the western blot results. Our staining results showed that compared to that in the negative control group, NOX4 protein was highly expressed in the lung fibroblast lesions of mice treated with PBS and naked PPGC NPs. The localization expression of NOX4 protein exhibited a clear cell localization specificity that matched the morphology of lung tissue. In the AS@PPGC NP, LIG@PPGC NP, and AS_LIG@PPGC NP groups, lung tissue showed a significant decrease in the NOX4-positive signal and area, and the AS_LIG@PPGC NP group showed the most significant decrease. The semiquantitative results showed significant differences among the different groups. Similarly, IHC staining of NLRP3 in lung tissue showed a trend that was consistent with that of NOX4 (Fig. 6H-K).

### Inhalation of AS/LIG/AS_LIG@PPGC NPs attenuates NOX4-p38 MAPK signaling pathway activation and proinflammatory cytokine levels

**Fig. 7:**
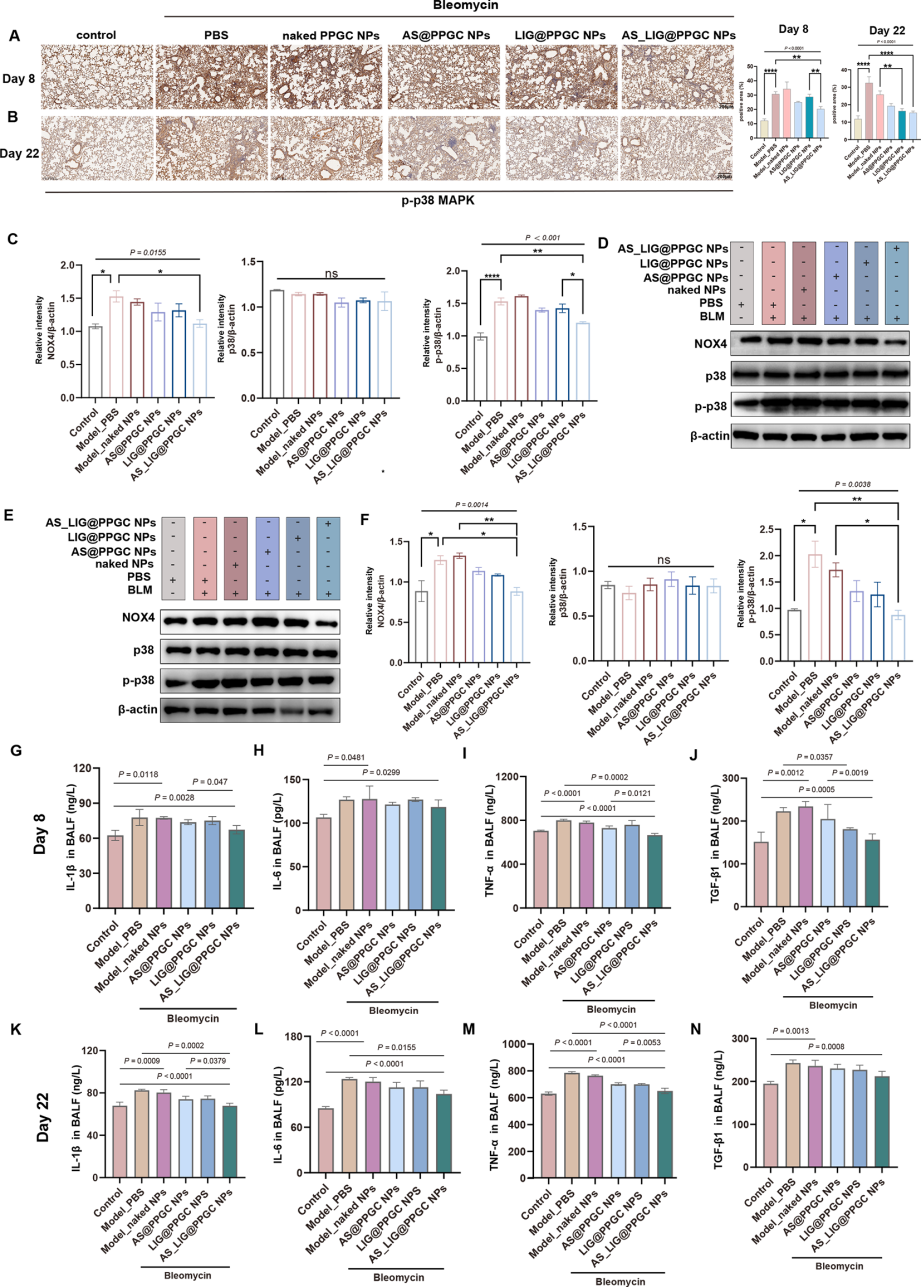
AS/LIG/AS_LIG@PPGC NPs inhalation reverses lung fibrosis caused by BLM through NOX4-p38 MAPK signalling pathway. (**A**, **B**). IHC staining analysis of lung sections and quantification of proteins related to NOX4-p38 MAPK signalling pathway on day 8, 22. Scale bar = 200 μm. (**A**). IHC staining analysis of lung sections of p-p38 MAPK. (**B**). Quantification of positive area of p-p38 MAPK. (**C**, **D**). Western blotting analysis of expression and quantification of proteins related to NOX4-p38 MAPK signalling pathway (NOX4, p38, and p-p38) in the lung sections on day 22 treated as indicated. (**E**, **F**). Western blotting analysis of expression and quantification of proteins related to NOX4-p38 MAPK signalling pathway (NOX4, p38, and p-p38) in the cells treated as indicated. (**G**-**K**). Expression levels of typical pro-inflammatory and profibrotic cytokines (TNF-α, IL-6, IL-1β, and TGF-β1) in BALF induced by BLM after different treatments at day 8 (**G**), and day 22 (**K**). The results were expressed as the mean ± SD (n = 3). * *P* < 0.05, **P < 0.01, ***P < 0.001, and ****P < 0.0001, n.s., not significant, *P* > 0.05

To investigate the role of the NOX4-p38 MAPK signaling pathway in IPF, we performed IHC staining to examine the phosphorylation levels of p38 MAPK (p-p38 MAPK) in the lung tissue in each group. The staining results revealed a significant increase in the p-p38 MAPK in model mice treated with PBS or naked NPs compared to those in the negative control group. In contrast, after treatment with AS@PPGC NPs, LIG@PPGC NPs, and AS_LIG@PPGC NPs, the level of p-p38 MAPK in the lung tissue of IPF model mice was decreased (Fig. 7A, B). Western blotting analysis of lung tissue samples showed similar results (Fig. 7C, D). Furthermore, compared to those in untreated cells, the protein levels of NOX4 and p-p38 MAPK were increased in cells treated with TGF-β1. However, treatment with AS@PPGC NPs, LIG@PPGC NPs, and AS_LIG@PPGC NPs decreased the protein levels of NOX4 and p-p38 MAPK in cells compared to those treated with PBS or naked NPs (Fig. 7E, F).

To further investigate the impact of AS/LIG/AS_LIG@PPGC NPs on pulmonary inflammation and fibrosis in IPF, we collected mouse BALF on Days 8 and 22 and measured the levels of IL-1β, IL-6, TNF-α, and TGF-β1 using ELISA. Our results demonstrated that the levels of these proinflammatory and profibrotic cytokines were significantly increased after BLM induction. However, compared to those in the groups treated with PBS or naked NPs, inhalation of AS@PPGC NPs, LIG@ PPGC NPs, and AS_LIG@PPGC NPs significantly decreased the levels of these cytokines in BALF (Fig. 7G-N). These results showed that AS/LIG/AS_LIG@PPGC NPs could modulate the NOX4/NLRP3/p38 MAPK signaling pathway in IPF, thereby alleviating pulmonary inflammation and fibrosis levels.

### RNA-seq analysis

**Fig. 8:**
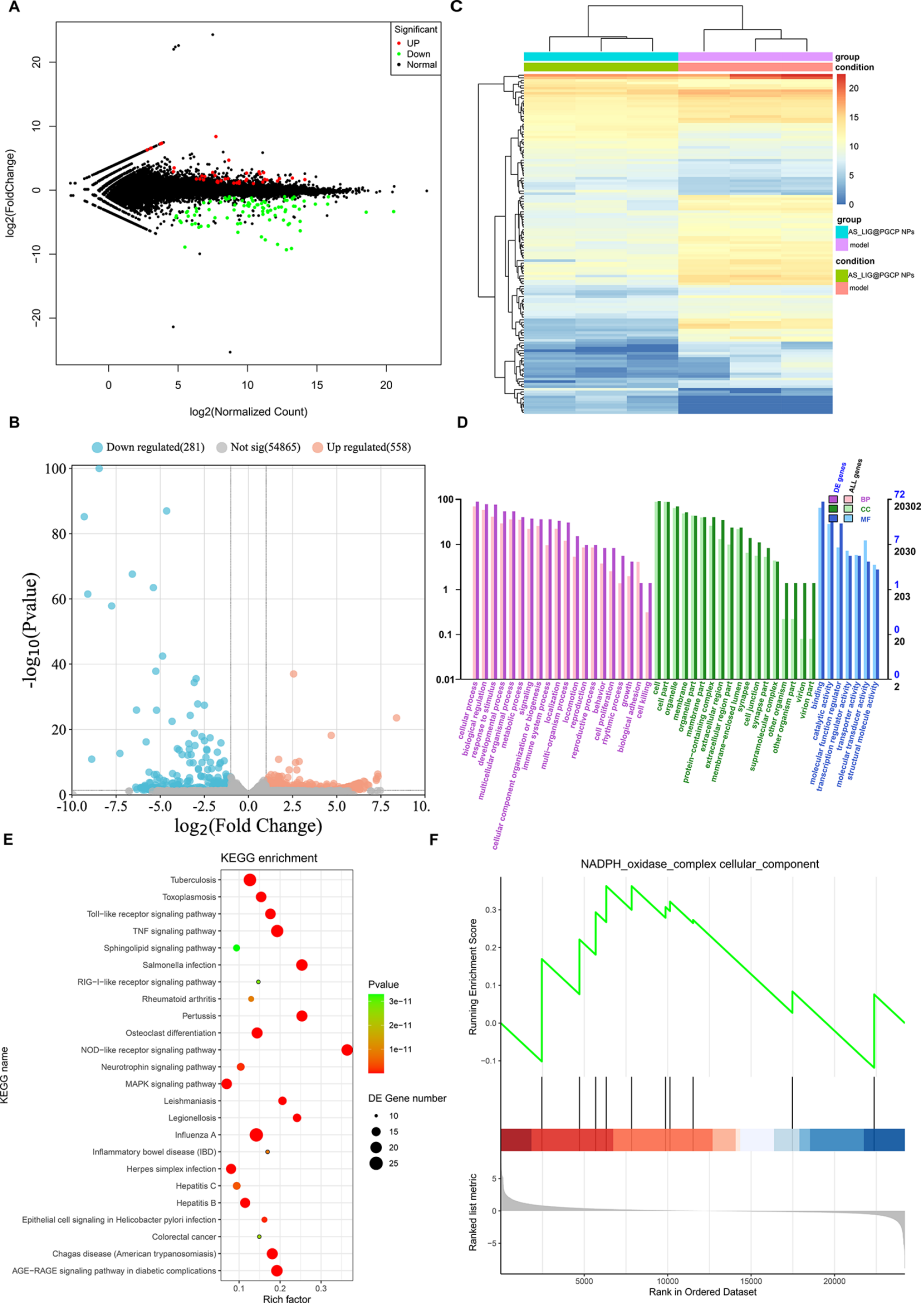
RNA-seq analysis. (**A**, **B**). Volcano plot of DE genes in transcription phase. The cutoff was set as log_2_(fold change) > 1 and adjusted *p* value < 0.05. The fold changes and *p* values were calculated by DESeq2. (**C**). Heatmap of the expression of the DE genes associated with model, AS_LIG@ PPGC groups. (**D**). The gene sets that were significantly enriched are arranged into categories of biological processes, cellular components, and molecular functions, and are presented in a bar plot. (**E**). Bubble plot of the KEGG pathway analysis enriched by differentially expressed genes between the model and AS_LIG@PPGC NPs groups. The colors of the nodes reflect the p-values of the designated pathways, and the sizes of the nodes indicate the number of differentially expressed genes enriched in the pathways. (**F**). GSEA analysis plot based on differentially expressed genes between the model and AS_LIG@PPGC NPs groups

Based on the therapeutic effect of AS/LIG/AS_LIG@PPGC NPs on IPF, we found that the antifibrotic effect of AS_LIG@PPGC NPs was the most significant. Therefore, we further investigated the underlying mechanism by RNA-seq analysis of mouse lung tissue. We identified differentially expressed genes (DE genes) between the model group and the AS_LIG@PPGC NPs group using two thresholds: log2 (fold change) ≥ 2 and FDR-adjusted *P* < 0.05. The results showed that 779 genes were significantly differentially expressed in the lung tissue of the AS_LIG@PPGC NPs group, including 558 upregulated genes and 221 downregulated genes compared to the model group (Fig. 8A, B). The heatmap (Fig. 8C) showed that the model group and the AS_LIG@PPGC NPs group were well separated at the gene level. We performed GO and KEGG enrichment analyses (Fig. 8D, E) to explore the biological processes and signaling pathways associated with the differentially expressed genes. Compared to the model group, the AS_LIG@PPGC NPs group showed significant enrichment in biological processes and functional pathways that dominate the improvement of IPF mechanisms. These enriched pathways affect the progression of IPF and involve biological processes, cellular structures, and molecular functions. The top 10 enriched pathways included oxidative stress, the inflammatory response, and immune system activity. In addition, we performed gene set enrichment analysis (GSEA) and found that the NADPH pathway was significantly enriched during the treatment of IPF with AS_LIG@PPGC NPs (Fig. 8F). This finding indicated that AS_LIG@PPGC NPs may exert their therapeutic effects on IPF by regulating the NADPH pathway. Our RNA-seq analysis results and the consistency with our in vitro and in vivo signaling pathway experiments provide evidence for the involvement of the NOX4/NLRP3/p38 MAPK signaling pathway in the therapeutic effect of AS_LIG@PPGC NPs on IPF.

## Discussion

The pathogenesis of IPF is quite complex and involves environmental factors, genetics, inflammation, and oxidative stress [9]. Among the detected biological processes, inflammation and oxidative stress are widely recognized as the core pathological mechanisms [1, 2]. In this study, the therapeutic effect of inhaled AS/LIG/AS_LIG@PPGC NPs on lung injury and PF induced by BLM was investigated. In addition, the potential therapeutic mechanisms by which by AS-IV and LIG inhibit inflammation and oxidative stress were validated. Our results demonstrated that inhalation of AS/LIG/AS_LIG@PPGC NPs significantly improved lung injury and pulmonary fibrosis, providing a new avenue for further investigation of their therapeutic mechanism.

Based on the theoretical foundation of the TCM approach of ‘tonifying Qi and activating blood circulation’ for in the treatment of IPF, we used a combination of AS-IV and LIG in our study. Our findings demonstrated that the inhalation of AS/LIG/AS_LIG@PPGC NPs could effectively mitigate lung injury and fibrosis induced by BLM. Further in vitro studies confirmed that this intervention significantly inhibited the formation of myofibroblasts, which are known to contribute to the development and progression of IPF. The BLM-induced IPF model has been widely used and established and can simulate the pathological process of human IPF [28]. It has been found that 7 days after BLM induction was the early stage of acute inflammation, which gradually transitioned into the fibrotic stage after 7 days [29]. To investigate the effect of AS/LIG/AS_LIG@PPGC NP inhalation on acute lung injury and fibrosis, we used a staged (Day 8 and Day 22) intervention and sampling approach. In this study, we observed that the BLM-induced mouse model of fibrosis exhibited characteristics similar to human IPF, including a marked increase in the number of inflammatory cells, thickening of alveolar walls, disordered alveolar structure, and substantial deposition of extracellular matrix (ECM). Various risk factors can induce local damage to alveolar epithelial cells, triggering abnormal communication between the epithelium and fibroblasts. Activated lung fibroblasts produce matrix myofibroblasts, leading to the accumulation of large amounts of ECM in the lung interstitial and ultimately causing lung structural remodeling, which is a recognized pathological process of IPF [30]. Furthermore, we demonstrated that inhalation of AS/LIG/AS_LIG@PPGC NPs had the significant antifibrotic effects, as evidenced by the marked reduction in the expression of α-SMA, COL1A1, and FN. These factors are typical markers of fibrosis, and their elevation often indicates myofibroblast activation, ECM deposition, and collagen fiber proliferation. Furthermore, we observed a significant reduction in the levels of TGF-β1 in the BALF. TGF-β1 has been shown to be both necessary and sufficient for the development of lung fibrosis [31]. Signaling mediated by TGF-β1 in epithelial and fibroblasts is a common and critical feature of fibrosis. In human IPF and BLM-induced pulmonary fibrosis models, TGF-β1 localized to sites of collagen deposition, and its expression preceded the deposition of collagen by a considerable margin [32]. These findings indicated that inhalation therapy can significantly alleviate the level of pulmonary fibrosis.

Controlling early pulmonary inflammation may be a prerequisite for achieving widely accepted antifibrotic effects. We observed that in the early stage of lung injury, the mouse model exhibited a collapsed lung structure, thickened alveolar walls, and robust infiltration of inflammatory cells. However, after inhaling AS/LIG/AS_LIG@PPGC NPs, there were significant reductions in the number of inflammatory cells in alveoli and the levels of inflammatory cytokines (IL-1β, IL-6, TNF-α) in BALF. Inflammation plays an important role in this process [1]. The activation of inflammatory cells and their factors can synergistically stimulate the proliferation of myofibroblasts and the secretion of pathological ECM. Therefore, inhibiting inflammation and blocking the activation of lung fibrosis cells to induce myofibroblasts are important measures to reduce the accumulation of ECM and structural remodeling. We were pleasantly surprised to discover that among the three drug-loaded NPs, AS_LIG@PPGC NPs exhibited the most significant anti-inflammatory and antifibrotic effects, indicating a good synergistic interaction between AS-IV and LIG. These findings further confirmed the advantages of the TCM concept of “tonifying Qi and promoting blood circulation”. They also provided scientific evidence and support for the use of TCM in the treatment of IPF. Importantly, these findings emphasize the necessity of further investigating the specific mechanisms of AS/LIG/AS_LIG@PPGC NPs.

NOX4-derived ROS and NLRP3 inflammasomes significantly contribute to the pathogenesis and progression of fibrosis. There is substantial evidence that NOX enzymes are present in various models of induced fibrosis, and NOX4 plays a critical role in the development of pulmonary fibrosis [33–35]. Multiple studies have consistently demonstrated that upregulation of NOX4 protein expression is involved in the fibrotic response induced by TGF-β. It facilitates fibrosis by enhancing oxidative stress through increased ROS production, activating myofibroblasts, and promoting extracellular matrix deposition [36–38]. Notably, NOX4-derived ROS are essential for BLM-induced pulmonary fibrosis, and they not only damage airway epithelial cells (AECs) but also participate in downstream fibrotic signaling pathways, such as the TGF-β1/SMAD pathway. Moreover, NOX-derived ROS are necessary for NLRP3 inflammasome activation. NOX-derived ROS can promote the binding of NLRP3 inflammasome by directly oxidizing thioredoxin-interacting protein (TXNIP). Additionally, it can indirectly affect the activation of NLRP3 inflammasome by modulating intracellular calcium ion concentration, mitochondrial function, and cellular environment [39]. The activation of NLRP3 inflammasomes promotes the activation of IL-18 and IL-1β, which subsequently promote fibrosis. It is worth noting that the activation of ROS also increases NOX4 production [40–42], which adds complexity to the relationship between NOX4-derived ROS and NLRP3 inflammasomes. In addition, the ROS generated by NOX4 can activate the p38 MAPK pathway, thereby triggering oxidative stress and inflammatory crosstalk reactions, ultimately leading to fibrosis [33, 36, 43]. NOX-derived ROS can directly oxidize and activate upstream regulatory factors, such as mitogen-activated protein kinase kinase kinase (MAPKKK), thereby indirectly activating the p38 MAPK signaling pathway. Alternatively, NOX-derived ROS can directly oxidize and activate p38 MAPK itself, altering the oxidation state of cysteine residues in p38 MAPK, leading to its activation [44]. Thus, the interaction between NOX4-derived ROS, NLRP3 inflammasome activation, and p38 MAPK constitutes a vicious cycle in pulmonary fibrogenesis, which also accounts for the ineffectiveness of single-target or single-drug interventions. While nintedanib, which is a tyrosine kinase inhibitor, has shown multitargeted efficacy in the treatment of IPF, its primary therapeutic effect involves its anti-inflammatory properties [45]. Furthermore, targeting all known fibrotic pathways could result in adverse effects and toxicity. Thus, taking a TCM approach, we chose two frequently used Qi-blood tonifying combinations and provided initial evidence for the substantial benefits of their combined application.

As effective bioactive components of *A. membranaceus* and *L. chuanxiong*, these factors have similar positive pharmacological activities, including anti-inflammatory, antioxidant, and immunoregulatory effects [44–46]. Existing evidence has demonstrated that AS-IV exerts antifibrotic effects through multiple signaling pathways in various fibrotic diseases, including TGF-β1/SMAD, p38 MAPK, NLRP3 inflammasomes, NOX4/p38 MAPK, and others [16–18, 47]. Similarly, LIG exerts antifibrotic effects through TGF-β1/SMAD and ERK/p38 MAPK, although most studies have been conducted on liver fibrosis [14, 15, 48]. Some studies have pointed out that AS-IV exerts antifibrotic effects by inhibiting NADPH oxidase-derived ROS [19]. Based on previous evidence, we chose AS-IV and LIG as promising drug candidates and hypothesized that they could exert their therapeutic effects by regulating the NOX4-ROS-p38 MAPK and NOX4-NLRP3 pathways to treat and prevent IPF. Encouragingly, our molecular docking results confirmed this hypothesis and showed that AS-IV could dock to the pockets of both NOX4 and NLRP3 proteins, while LIG could dock to the pocket of NLRP3 protein. These results prompted us to further explore and investigate these compounds. Therefore, we conducted in vitro and in vivo studies to further validate our findings.

Targeting NOX4-derived ROS and disrupting the self-perpetuating loop between NOX4, ROS, and NLRP3 inflammasomes are potential strategies for intervening in the pathogenesis and progression of pulmonary fibrosis. Consistent with previous research findings, Sun et al. reported that MenSC-Exo treatment improved BLM-induced pulmonary fibrosis by inhibiting ROS and NLRP3 inflammasome activation [36]. Our study demonstrated that AS/LIG/AS_LIG@PPGC NPs possessed antifibrotic properties by inhibiting the NOX4-ROS-p38 MAPK and NOX4-NLRP3 signaling pathways. First, our in vitro experiments demonstrated that AS/LIG/AS_LIG@PPGC NPs significantly reduced intracellular ROS levels, as evidenced by decreased ROS fluorescence intensity measured using DCFH-DA. Furthermore, our in vivo and in vitro experiments, which used methods such as western blotting, IHC staining, and immunofluorescence, confirmed the significant upregulation of NOX4, NLRP3, p-p38, and related pathways in the lungs of mice with BLM-induced pulmonary fibrosis and the significant inhibition of this upregulation following treatment with AS/LIG/AS_LIG@PPGC NPs.

Although the present study used a mouse nebulization model to investigate IPF, there were several limitations to this approach. Mice and humans differ in lung anatomy, respiratory rate, and lung ventilation, and their respiratory systems may respond differently to certain drugs. Therefore, the mouse nebulization model cannot fully replace human studies. Nevertheless, our study showed that targeting NOX4-generated ROS and disrupting the self-sustaining cycle between NOX4, ROS, and NLRP3 inflammasomes could effectively inhibit the occurrence and progression of pulmonary fibrosis. Additionally, our research identified the potential application value of AS/LIG/AS_LIG@PPGC NPs in the treatment of IPF. Future studies can explore the pharmacokinetics and toxicity of AS/LIG/AS_LIG@PPGC NPs to improve their application in the treatment of IPF.

## Conclusion

In summary, our study demonstrated that inhalation therapy is a feasible and unique strategy for treating lung injury and IPF. PPGC NPs, serving as drug carriers, are well-suited for pulmonary inhalation therapy. Our in vitro and in vivo experiments showed that AS/LIG/AS_LIG@PPGC NPs can exert significant antifibrotic effects by downregulating the NOX4-ROS-p38 MAPK and NOX4-NLRP3 signaling pathways, reducing ROS production, and breaking the vicious cycle between NOX4 and NLRP3 inflammasomes. Furthermore, AS_LIG@PPGC NPs exhibited the most significant therapeutic effect, highlighting the good combination of TCM and modern medicine. The TCM theory of “tonifying qi and activating blood” has broad application prospects for the treatment of lung fibrosis and injury. The use of AS/LIG/AS_LIG@PPGC NPs, which is a potential therapeutic strategy, hold significant clinical value. Future research can further explore the therapeutic mechanism of AS/LIG/AS_LIG@PPGC NPs, optimize drug design to achieve better therapeutic effects, and develop more integrated therapies based on traditional Chinese and Western medicine.

### Electronic supplementary material

Below is the link to the electronic supplementary material.

Supplementary Material 1

## Data Availability

Not applicable.

## References

[1] Bringardner BD, Baran CP, Eubank TD, Marsh CB (2008). The role of inflammation in the pathogenesis of Idiopathic Pulmonary Fibrosis. Antioxid Redox Signal.

[2] Otoupalova E, Smith S, Cheng G, Thannickal VJ (2020). Oxidative stress in Pulmonary Fibrosis. Compr Physiol.

[3] Kolahian S, Fernandez IE, Eickelberg O, Hartl D (2016). Immune mechanisms in Pulmonary Fibrosis. Am J Respir Cell Mol Biol.

[4] Geng J, Liu Y, Dai H, Wang C (2021). Fatty acid metabolism and Idiopathic Pulmonary Fibrosis. Front Physiol.

[5] Salvati L, Palterer B, Parronchi P (2020). Spectrum of Fibrotic Lung Diseases. N Engl J Med.

[6] Duchemann B, Annesi-Maesano I, Jacobe de Naurois C, Sanyal S, Brillet P-Y, Brauner M (2017). Prevalence and incidence of interstitial lung Diseases in a multi-ethnic county of Greater Paris. Eur Respir J.

[7] Raghu G, Chen S-Y, Hou Q, Yeh W-S, Collard HR (2016). Incidence and prevalence of Idiopathic Pulmonary Fibrosis in US adults 18–64 years old. Eur Respir J.

[8] Maher TM, Bendstrup E, Dron L, Langley J, Smith G, Khalid JM (2021). Global incidence and prevalence of Idiopathic Pulmonary Fibrosis. Respir Res.

[9] Raghu G, Remy-Jardin M, Richeldi L, Thomson CC, Inoue Y, Johkoh T (2022). Idiopathic Pulmonary Fibrosis (an update) and Progressive pulmonary fibrosis in adults: an Official ATS/ERS/JRS/ALAT Clinical Practice Guideline. Am J Respir Crit Care Med.

[10] Lancaster LH, de Andrade JA, Zibrak JD, Padilla ML, Albera C, Nathan SD (2017). Pirfenidone safety and adverse event management in Idiopathic Pulmonary Fibrosis. Eur Respir Rev.

[11] Anonymous (2015). Efficacy and safety of Nintedanib in Idiopathic Pulmonary Fibrosis. N Engl J Med.

[12] Yin Z-F, Wei Y-L, Wang X, Wang L-N, Li X (2020). Buyang Huanwu Tang inhibits cellular epithelial-to-mesenchymal transition by inhibiting TGF-β1 activation of PI3K/Akt signaling pathway in pulmonary fibrosis model in vitro. BMC Complement Med Ther.

[13] Chen H, Song H, Liu X, Tian J, Tang W, Cao T (2017). Buyanghuanwu Decoction alleviated pressure overload induced cardiac remodeling by suppressing Tgf-β/Smads and MAPKs signaling activated fibrosis. Biomed Pharmacother.

[14] Zhang F, Ni C, Kong D, Zhang X, Zhu X, Chen L (2012). Ligustrazine attenuates oxidative stress-induced activation of hepatic stellate cells by interrupting platelet-derived growth factor-β receptor-mediated ERK and p38 pathways. Toxicol Appl Pharmacol.

[15] Qiu J-L, Zhang G-F, Chai Y-N, Han X-Y, Zheng H-T, Li X-F (2022). Ligustrazine attenuates Liver Fibrosis by Targeting miR-145 mediated transforming growth Factor-β/Smad signaling in an animal model of biliary atresia. J Pharmacol Exp Ther.

[16] Li X, Wang X, Han C, Wang X, Xing G, Zhou L (2013). Astragaloside IV suppresses collagen production of activated hepatic stellate cells via oxidative stress-mediated p38 MAPK pathway. Free Radic Biol Med.

[17] Qian W, Cai X, Qian Q, Zhang W, Wang D (2018). Astragaloside IV modulates TGF-β1-dependent epithelial-mesenchymal transition in bleomycin-induced pulmonary fibrosis. J Cell Mol Med.

[18] Li N, Wu K, Feng F, Wang L, Zhou X, Wang W (2021). Astragaloside IV alleviates silica–induced pulmonary fibrosis via inactivation of the TGF–β1/Smad2/3 signaling pathway. Int J Mol Med.

[19] Lin J, Fang L, Li H, Li Z, Lyu L, Wang H (2019). Astragaloside IV alleviates doxorubicin induced cardiomyopathy by inhibiting NADPH oxidase derived oxidative stress. Eur J Pharmacol.

[20] Chen Y-B, Zhang Y-B, Wang Y-L, Kaur P, Yang B-G, Zhu Y (2022). A novel inhalable quercetin-alginate nanogel as a promising therapy for acute lung injury. J Nanobiotechnol.

[21] Rasooli R, Rajaian H, Pardakhty A, Mandegary A (2018). Preference of aerosolized pirfenidone to oral intake: an experimental model of Pulmonary Fibrosis by Paraquat. J Aerosol Med Pulm Drug Deliv.

[22] Laube BL, Janssens HM, de Jongh FHC, Devadason SG, Dhand R, Diot P (2011). What the pulmonary specialist should know about the new inhalation therapies. Eur Respir J.

[23] Brown JS, Gordon T, Price O, Asgharian B (2013). Thoracic and respirable particle definitions for human health risk assessment. Part Fibre Toxicol.

[24] Hill DB, Button B, Rubinstein M, Boucher RC (2022). Physiology and pathophysiology of human airway mucus. Physiol Rev.

[25] Bowden DH (1984). The alveolar macrophage. Environ Health Perspect.

[26] Edwards DA, Hanes J, Caponetti G, Hrkach J, Ben-Jebria A, Eskew ML (1997). Large porous particles for pulmonary drug delivery. Science.

[27] Patel B, Gupta N, Ahsan F (2015). Particle engineering to enhance or lessen particle uptake by alveolar macrophages and to influence the therapeutic outcome. Eur J Pharm Biopharm.

[28] Chua F, Gauldie J, Laurent GJ (2005). Pulmonary fibrosis: searching for model answers. Am J Respir Cell Mol Biol.

[29] Kolb P, Upagupta C, Vierhout M, Ayaub E, Bellaye PS, Gauldie J (2020). The importance of interventional timing in the bleomycin model of pulmonary fibrosis. Eur Respir J.

[30] Richeldi L, Collard HR, Jones MG (2017). Idiopathic Pulmonary Fibrosis. Lancet.

[31] Kim KK, Sheppard D, Chapman HA (2018). TGF-β1 signaling and tissue fibrosis. Cold Spring Harb Perspect Biol.

[32] Hoyt DG, Lazo JS (1988). Alterations in pulmonary mRNA encoding procollagens, fibronectin and transforming growth factor-beta precede bleomycin-induced pulmonary fibrosis in mice. J Pharmacol Exp Ther.

[33] Crestani B, Besnard V, Boczkowski J (2011). Signalling pathways from NADPH oxidase-4 to Idiopathic Pulmonary Fibrosis. Int J Biochem Cell Biol.

[34] De Minicis S, Brenner DA (2007). NOX in liver fibrosis. Arch Biochem Biophys.

[35] Lambeth JD, Krause K-H, Clark RA (2008). NOX enzymes as novel targets for drug development. Semin Immunopathol.

[36] Hao B, Sun R, Guo X, Zhang L, Cui J, Zhou Y (2021). NOX4-Derived ROS promotes collagen I deposition in bronchial smooth muscle cells by activating Noncanonical p38MAPK/Akt-Mediated TGF-β signaling. Oxid Med Cell Longev.

[37] Chan EC, Peshavariya HM, Liu G-S, Jiang F, Lim S-Y, Dusting GJ (2013). Nox4 modulates collagen production stimulated by transforming growth factor β1 in vivo and in vitro. Biochem Biophys Res Commun.

[38] Hecker L, Vittal R, Jones T, Jagirdar R, Luckhardt TR, Horowitz JC (2009). NADPH oxidase-4 mediates myofibroblast activation and fibrogenic responses to lung injury. Nat Med.

[39] Pfeiffer ZA, Guerra AN, Hill LM, Gavala ML, Prabhu U, Aga M (2007). Nucleotide receptor signaling in murine macrophages is linked to reactive oxygen species generation. Free Radic Biol Med.

[40] Noguchi T, Ishii K, Fukutomi H, Naguro I, Matsuzawa A, Takeda K (2008). Requirement of reactive oxygen species-dependent activation of ASK1-p38 MAPK pathway for extracellular ATP-induced apoptosis in macrophage. J Biol Chem.

[41] Cruz CM, Rinna A, Forman HJ, Ventura ALM, Persechini PM, Ojcius DM (2007). ATP activates a reactive oxygen species-dependent oxidative stress response and secretion of proinflammatory cytokines in macrophages. J Biol Chem.

[42] Wu M, Xing Q, Duan H, Qin G, Sang N (2022). Suppression of NADPH oxidase 4 inhibits PM2.5-induced cardiac fibrosis through ROS-P38 MAPK pathway. Sci Total Environ.

[43] Liu F, Bayliss G, Zhuang S (2019). Application of nintedanib and other potential anti-fibrotic agents in fibrotic Diseases. Clin Sci (Lond).

[44] Lin J, Wang Q, Zhou S, Xu S, Yao K (2022). Tetramethylpyrazine: a review on its mechanisms and functions. Biomed Pharmacother.

[45] Zhu Y, Chai Y, Xiao G, Liu Y, Xie X, Xiao W (2022). Astragalus and its formulas as a therapeutic option for fibrotic Diseases: Pharmacology and mechanisms. Front Pharmacol.

[46] Gong F, Qu R, Li Y, Lv Y, Dai J (2022). Astragalus Mongholicus: a review of its anti-fibrosis properties. Front Pharmacol.

[47] Wan Y, Xu L, Wang Y, Tuerdi N, Ye M, Qi R (2018). Preventive effects of astragaloside IV and its active sapogenin cycloastragenol on cardiac fibrosis of mice by inhibiting the NLRP3 inflammasome. Eur J Pharmacol.

[48] Wu X, Zhang F, Xiong X, Lu C, Lian N, Lu Y (2015). Tetramethylpyrazine reduces inflammation in liver fibrosis and inhibits inflammatory cytokine expression in hepatic stellate cells by modulating NLRP3 inflammasome pathway. IUBMB Life.

