# Supplementary material for: Traditional Chinese medicine inspired dual-drugs loaded inhalable nano-therapeutics alleviated idiopathic pulmonary fibrosis by targeting early inflammation and late fibrosis
Source: J Nanobiotechnology. 2024 Jan 3;22:14. doi: 10.1186/s12951-023-02251-0 (PMC10763202; doi:10.1186/s12951-023-02251-0)
Supplement: Supplementary file 1 — Supplementary Material 1 [file 12951_2023_2251_MOESM1_ESM.docx]

# Supporting Information

**Traditional Chinese Medicine Inspired Dual-drugs Loaded Inhalable Nano-therapeutics Alleviated Idiopathic Pulmonary Fibrosis by Targeting Early Inflammation and Late Fibrosis**

Meiling Zheng^1,2^ Kai Liu^4^ Lei Li^5*^ Cuiling Feng^1,2*^ Guanghao Wu^3*^

^1^ Dongzhimen Hospital, Beijing University of Chinese Medicine, Beijing, 100010, China.

^2^ Peking University People’s Hospital, Beijing, 100032, China.

^3^ School of Materials Science & Engineering, Beijing Institute of Technology, Beijing, 100081, China.

^4^ Division of Pulmonary and Critical Care Medicine, Kunming Children’s Hospital, Kunming, 650000, China.

^5^ National Clinical Research Center for Obstetric & Gynecologic Diseases, Department of Obstetrics and Gynecology, Peking Union Medical College Hospital, Chinese Academy of Medical Sciences & Peking Union Medical College, Beijing, 100032, China.

^*^Corresponding author: Lei Li, E-mail: [lilei64@pumch.cn](mailto:lilei64@pumch.cn). Culling Feng, E-mail: [fengcuiling@sina.com](mailto:fengcuiling@sina.com). Guanghao Wu, E-mail: [bityswgh@bit.edu.cn](mailto:bityswgh@bit.edu.cn).

**Figure S1 Size of AS_LIG@PPGC NPs within 5 days.**

**Figure S2 TEM images before and after the inhalation of AS_LIG@PPGC NPs. (A). TEM images before inhalation. (B). TEM images after inhalation.**

**Figure S3. Evaluation of the biocompatibility of nanoformulations in vivo (heart, liver, spleen, and kidney). Scale bar = 200 µm.**

**Figure S4. RNA-Seq analysis between model group and AS_LIG@PPGC NPs group.** (A). GO enrichment in the biological processes. (B). GO enrichment in the cellular component annotations. (C). GO enrichment in the mammalian phenotypes. (D). classification system used in the KEGG database.

**Figure S5.** Distribution of NPs inhalation in different lung lobes (right upper lobe, middle lobe, lower lobe, and left lobe) at various time points (0h, 2h, 4h, 6h, 12h, 24h). Scale bar = 200 μm
